# Biodegradable collagen matrix (Ologen™) implant and conjunctival autograft for scleral necrosis after pterygium excision: two case reports

**DOI:** 10.1186/s12886-015-0130-z

**Published:** 2015-10-24

**Authors:** Chan-Ho Cho, Sang-Bumm Lee

**Affiliations:** Wolhang Public Health Center, # 1151, Anpo-ri, Wolhang-myeon, Seongju-gun, Gyeongsang-bukdo 719-851 South Korea; Department of Ophthalmology, Yeungnam University College of Medicine, #170, Hyunchung-ro, Nam-gu, Daegu, 705-717 South Korea

**Keywords:** Mitomycin C, Ologen collagen matrix, Pterygium excision, Scleral necrosis, Scleromalacia

## Abstract

**Background:**

Scleromalacia, in the form of scleral thinning, melting, and necrosis, is a potentially serious complication of pterygium excision. This study introduces a new biodegradable material, Ologen™ collagen matrix (OCM), to repair scleral thinning as an alternative to preserved scleral tissue, and evaluates the long-term outcomes of OCM for ocular surface reconstruction surgery.

**Case presentation:**

Two cases of possibly mitomycin C (MMC)-associated marked scleral thinning after pterygium excision with 0.02 % topical MMC for 2-weeks were included in this study. An OCM graft at the scleral thinning area and conjunctival autograft (CAU) were performed on both patients. The scleral defect size was measured and its margin was marked with a biopsy punch. The margin of the scleral thinning area was trimmed by Vannas scissors and the OCM was cut using a circular-shape biopsy punch of the same size. The OCM was sutured with a recipient scleral wall using 10–0 nylon interrupted sutures. Free CAU was harvested from the superonasal bulbar conjunctiva with a punch biopsy 1-mm larger in diameter than that of the OCM. The previously sutured OCM bed was covered with CAU and the graft was secured with 10–0 nylon interrupted sutures. Both patients were examined periodically for over two years by assessing graft thickness and surface vascularization using a slit lamp biomicroscope. Reepithelialization of the ocular surface was observed within three to six days after surgery. Ocular discomfort and inflammation ceased in both patients as the ocular surface quickly stabilized. The entire graft site remained intact and provided a good healthy ocular surface with fluorescein stain negative intact epithelium and good vascularization of grafted conjunctiva. Epithelial defects and scleral thinning did not recur in either patient over the two year follow-up period.

**Conclusion:**

For treatment of a possibly MMC-associated scleral necrosis following the surgical excision of the pterygium, an OCM graft with CAU is highly recommended for good clinical outcomes and low recurrence rates. With the clinical results of this study, the new biodegradable Ologen™ collagen matrix qualifies as an alternative treatment to scleral tissue for ocular surface reconstruction.

## Background

Scleromalacia in the form of scleral thinning, melting, and necrosis is a potentially serious complication of pterygium excision. Scleromalacia has been observed after bare sclera excision alone and may occur years or even decades after the original surgery. The risk of scleromalacia seems to be exacerbated by adjunctive therapies such as β-irradiation, thiotepa, and mitomycin C (MMC) [1, 2]. Scleral necrosis occurs in 0.2 to 4.5 % of patients, and higher risk is linked to adjunctive use of MMC, especially more concentrated or repeated doses [3].

If scleral thinning is severe and progressive, it can cause visual loss from secondary infection and compromised tectonic disintegrity of the globe by protrusion of choroidal tissue even with minor trauma. Once an infection develops, it is extremely difficult to control and may lead to the most devastating complication, infectious scleritis. Therefore, prevention of the catastrophic infection is more important than treatment, and the ocular surface reconstructive surgery is required for therapeutic or tectonic reasons. The technique of scleral patching with conjunctiva involves mobilization of a flap of conjunctiva, with or without attached Tenon’s fascia, and suturing of the flap over the scleral defect. Other techniques include use of tarsoconjunctival flap with autologous or homologous sclera, fascia lata, tibial periosteum, split-thickness dermis, amniotic membrane, aorta, dura, pericardium, or synthetic materials for patch graft [4–6].

In this study, we used the biodegradable material, Ologen™ collagen matrix (OCM, Aeon Astron Europe B.V., Leiden, The Netherlands), implant as graft tissue [7]. OCM as an implant does not require donor preserved scleral tissue, the OCM is able to fill the space between the CAU and scleral bed easily, and it helps the ocular surface stay flat and regular. In this case study, we present results of two years of observation for clinical outcomes and complications of the OCM graft with CAU, and demonstrate its clinical usefulness.

## Case presentation

### Case 1

A 70-year-old woman was referred to our institution in July 2012 for treatment of left ocular pain. She complained of ocular discomfort and decreased visual acuity (12/20) at the first visit. She underwent nasal pterygium excision with topical MMC in the left eye six years previously at another facility. A few years after surgery, symptoms of intermittent ocular discomfort and mild ocular pain developed, so she went for treatment to a local medical center. She had neither medical history or family history of ophthalmic disease.

**Fig. 1:**
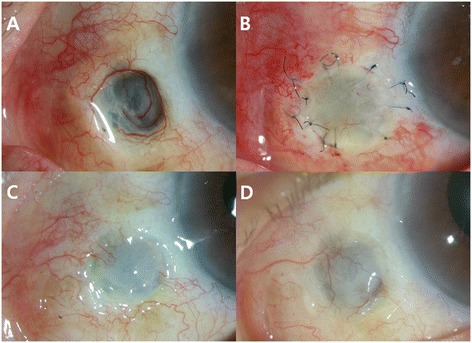
Preoperative and postoperative slit-lamp photographs of Case 1, the left eye. **a** Preoperative photography showing large severe scleral thinning and excavation with impending uveal exposure. **b** First week after surgery. **c** One month after surgery. **d** Two years after surgery

On slit-lamp examination, the underlying sclera at the site of the prior pterygium excision was necrotic and avascular, and showed marked thinning in the nasal portion of the left eye (Fig. 1a). The scleral bed appeared conjunctivalization over the exposed ciliary body with no evidence of scleral perforation sign by Seidel test. The adjacent corneal epithelium was intact and there was no anterior chamber inflammatory reaction. We thought that the scleral thinning was a possible MMC-associated thinning. Microbial smears and cultures of the scleral bed were obtained at the first visit, but were negative.

**Fig. 2:**
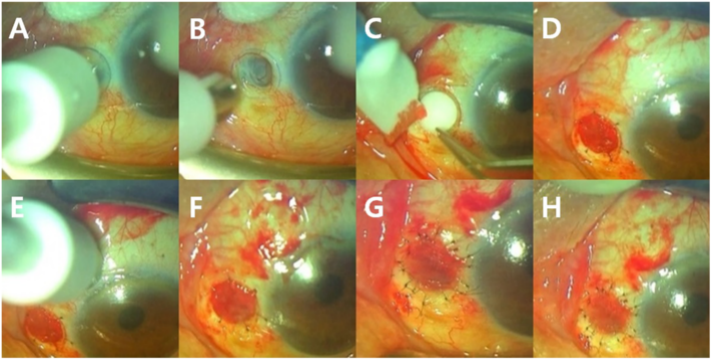
The surgical procedure for Ologen™ collagen matrix (OCM) graft and conjunctival autograft surgery. **a**,**b** The size of the scleral defect was measured and its margin was marked with a 3-mm diameter biopsy punch to determine the boundary of conjunctivectomy, **c** The margin of scleral thinning area was trimmed by Vannas scissors and the OCM was cut with a circular-shaped biopsy punch of the same size (3-mm diameter). The OCM was trimmed and fitted to cover the scleral defect. **d** The OCM was sutured with a recipient scleral wall using six stitches of 10–0 nylon interrupted sutures. The color of the OCM changed from white to red due to blood accumulation. **e** A 4-mm diameter, circular, free CAU was harvested from the superonasal bulbar conjunctiva with a punch biopsy 1 mm larger in diameter than that of the piece of OCM. **f** The CAU was carefully positioned over the previously sutured OCM bed. **g** The CAU was anchored to the scleral wall and the healthy conjunctival margin through the OCM bed with 11 stitches of interrupted sutures of the 10–0 nylon. **h** Immediate final postoperative appearance

The patient was started on topical 0.1 % fluorometholone and 0.5 % levofloxacin eye drops. Two weeks later, the patient’s ocular pain and discomfort was gone. After informed consent, the patient underwent an OCM graft with CAU at the scleral thinning area. The surgical technique is described in Fig. 2. Proparacaine hydrochloride eye drop (0.5 %, Alcaine; Alcon, Fort Worth, TX) was used as topical anesthesia before surgery. First, the necrotic soft tissues and devitalized sclera were surgically debrided with a diamond burr, taking care not to damage the exposed ciliary body and the unaffected adjacent conjunctiva. Gentle polishing with a diamond burr instead of a knife can produce a flat, regular surface without damaging the already much thinned scleral bed. All necrotic scleral tissue was dissected away until the scleral surface was clean, smooth, and even. Conjunctival hemorrhage during debridement was controlled by ocular bovie and cotton swab compression. To determine the boundary for the conjunctivectomy, the size of the scleral defect was measured and its margin was marked with a 3-mm diameter biopsy punch (Fig. 2a, b). The margin of scleral thinning area was trimmed by Vannas scissors and the OCM was cut with a circular-shaped biopsy punch of the same size (3-mm diameter). The OCM was trimmed and fitted to cover the scleral defect (Fig. 2c). The OCM was sutured with a recipient scleral wall using six stitches of 10–0 nylon interrupted sutures. The color of the OCM changed from white to red due to blood accumulation (Fig. 2d). Once the scleral defect was repaired, a 4-mm diameter, circular, free CAU was harvested from the superonasal bulbar conjunctiva with a punch biopsy 1 mm larger in diameter than that of the piece of OCM. A conjunctival graft that is larger than the scleral defect can achieve a stable, tension-free graft to avoid a wound dehiscence (Fig. 2e, f). The CAU was carefully positioned over the previously sutured OCM bed and anchored to the scleral wall and the healthy conjunctival margin through the OCM bed with 11 stitches of interrupted sutures of 10–0 nylon (Fig. 2g, h).

After surgery, the patient was given a patch dressing with a topical antibiotic ointment (Erythromycin, Ecolicin^®^, Taejoon Pharm., Seoul, Korea) and a steroid ointment (Dexamethasone, Maxitrol^®^, Alcon Laboratories Inc., Fort Worth, TX, USA) to be taken four times daily. The patient was examined daily for the first five postoperative days. After five days, the ointment patch dressing was replaced by topical antibiotics (0.5 % levofloxacin, Cravit^®^, Taejoon Pharm, Seoul, Korea) and steroid eye drops (0.1 % fluorometholone, Flarex^®^, Alcon Laboratories Inc., Fort Worth, TX, USA) to be taken four times a day. Inflammation of the scleral bed subsided and the patient was comfortable. The ocular surface was re-epithelialized six days after surgery. The conjunctival sutures were removed at intervals of one week to one month over the postoperative course. Starting about one month after surgery, the topical 0.1 % fluorometholone and 0.5 % levofloxacin eye drops were tapered off over three months. Artificial tear substitutes were used continuously after surgery. The patient was examined weekly for the first two months and at three, four, six, nine, 12, 16, 20, and 24 months postoperative.

Over the first postoperative month, the graft site vascularized and was taken up well (Fig. 1c). The conjunctival surface was stable at six months and 12 months postoperative. When reviewed 24 months later, the conjunctival surface was still stable with no recurrence of scleral thinning and scleromalacia (Fig. 1d).

### Case 2

A 76-year-old man visited our institution complaining of long standing ocular discomfort and decreased visual acuity (16/20). He underwent nasal pterygium excision with topical MMC in his left eye 10 years prior at another facility. He had neither medical history or family history of ophthalmic disease.

**Fig. 3:**
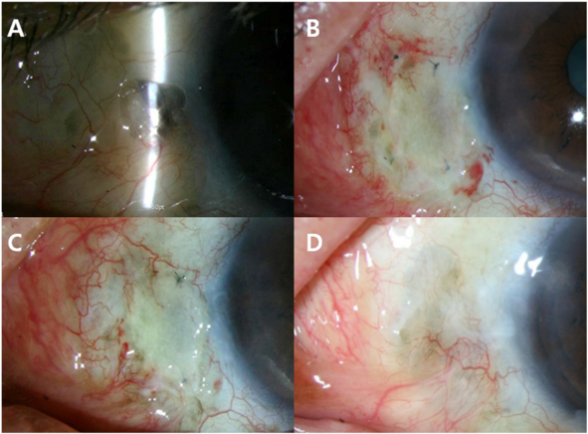
Preoperative and postoperative slit-lamp photographs of Case 2, the left eye. **a** Preoperative photography showing irregular scleral thinning and excavation with impending uveal exposure. **b** First week after surgery. **c** One month after surgery. **d** Two years after surgery

On slit-lamp examination, a focal epithelial defect at the site of the prior pterygium excision and an approximately 3 mm scleral excavation with impending uveal exposure were observed (Fig. 3a). At presentation, there was no sign of infectious scleromalacia. We thought that the scleral excavation was possibly an MMC-associated lesion. Microbial staining and cultures were negative.

In the same manner as described in Case 1, steroid and antibiotic eye drops were used before surgery. We performed an OCM graft with CAU. Postoperatively, the patient was treated with topical eye drops and examined daily for the first postoperative week. Three days after surgery, the OCM graft vascularized and was taken up well. The surrounding conjunctival and scleral inflammation subsided and the patient was comfortable. The reconstructed ocular surface was stable with no complications during the two-year follow-up period (Fig. 3b,c,d).

## Conclusions

A recently developed biodegradable porous collagen-glycosaminoglycan (GAG) copolymer matrix implant is proposed for glaucoma surgery because of its modulatory effect on wound healing [8–10].

The biodegradable collagen matrix implant marketed as Ologen™ is a novel bioengineered implant designed to be used during trabeculectomy [11]. The Ologen™ is an upgraded biodegradable, implantable scaffold collagen matrix consisting of lyophilized porcine atelocollagen (>90 %) and lyophilized porcine glycosaminoglycan (<10 %) with pore sizes of 10 to 300 μm. Atelocollagen is a highly purified pepsin-treated type I collagen. A collagen molecule has an amino acid sequence, a telopeptide, at both the N and C terminals, which confers most of the collagen’s antigenicity. Atelocollagen obtained by pepsin treatment is low in immunogenicity, because it is free of telopeptides [12].

Ologen™ is available in various shapes and dimensions. When Ologen™ is placed into the subconjunctival space, the porous structure guides conjunctival fibroblasts and myoblasts to proliferate randomly and secrete connective tissue in the form of a loose matrix during the wound healing process. Therefore, it reduces scar formation and wound contraction. Due to this mechanism, Ologen™ is being used to enhance wound healing in ocular surface reconstruction with low immunogenicity [9]. After Ologen™ implantation, the device completely degrades within 90–180 days. The implant leaves behind a loose alignment of collagen fibers inside scleral defects that is remarkably similar to normal tissue, with less scar formation than would otherwise have occurred [9]. These findings suggest that Ologen™ provides a physiological structure for tissue repair, inducing a conjunctival wound to heal more in a physiological than a pathological process. Therefore, this interventional case series study, using Ologen™ as graft tissue for scleral thinning and evaluating its long term outcomes, is significant.

Other patching materials including preserved sclera, amniotic membrane, and autologous fascia lata in scleral patching are good donor material but still have some limitations. For example, preserved homologous sclera is a flexible avascular tissue that causes minor immunologic reactions, infectious disease transmission, and potential barrier to fibrovascular ingrowth. Corneal tissue has a higher radius of curvature compared with sclera, and placement of a large corneal patch on the scleral bed usually results in a more raised lesion. Pericardium, fascia lata, and dura are hard to obtain and sometimes require additional surgery. Amniotic membrane is not suitable as space filling material [3]. Autologous scleral tissue grafts or partial thickness scleral flap rotational procedures can be used to patch scleral defects, although these procedures potentially can damage the scleral tissue harvesting site, especially in elderly patients like in this study. Therefore the authors decided to try the new biomaterial instead of autologous scleral tissue to avoid additional scleral or ocular surface damage from the surgical procedure itself. The Ologen™ material is mainly used in glaucoma filtering surgeries because its porous and spongy structure permits percolation of aqueous material during wound healing. The authors believe that the Ologen™ material cannot provide sufficient integrity, such as a tough-walled tunica sclerosa, if there is large scleral thinning with an underlying uveal protruding ectatic surface change. However, in the case of a small scleral excavation without any underlying uveal ectatic change, the porous, spongy properties of Ologen™ provide enough space filler effect for the duration of the healing process of the wound with connective tissue replacement.

In this study, we used the new biodegradable and implantable material, Ologen™ as graft tissues for scleral necrosis treatment after pterygium excision. In our cases, all of the Ologen™ graft with CAU remained intact and provided good healthy ocular surface; moreover, recurrence of epithelial defect and scleral thinning was not observed in either patient for over the two-year follow-up period.

Our study shows the Ologen™ collagen matrix to be a recommendable alternative material in ocular surface reconstruction surgery for repair of scleromalacia. Based on our results, the Ologen™ implant can induce host tissue-collagen matrix interaction, optimize and stabilize the structure, and prevent scar formation or further infection. Compared to scleral or other tissue graft, the Ologen™ graft is a time-saving, straightforward technique. As the Ologen™ material is readily affordable, the Ologen™ graft is a valuable technique for cases in which donor scleral tissue is unavailable or not accepted. Furthermore, we placed a conjunctival autograft over the OCM implant to produce complete epithelialization of the bare OCM surface while mitigating ocular surface inflammation and patient discomfort for the duration of the conjunctival epithelial migration. We expected the meticulous conjunctival autograft overlying the OCM implant to promote prompt epithelialization, vascularization, and wound remodeling of the conjunctival tissue.

There are some limitations to this study. First, quantitative measurements of postoperative scleral thickness and the integrity of the graft site using AS-OCT is needed in more cases. Second, we believe the OCM graft applied in this study is appropriate only for patients with a small scleral excavation and no underlying uveal ectactic change. Third, a larger number of participants will be required to assess the clinical usefulness of the OCM implant substitute for preserved sclera in scleral necrosis repair surgery. Despite these limitations, this study provides a new surgical technique with the application of new materials to scleral necrosis surgery. The two-year follow-up period was sufficient to evaluate the outcome of the surgery. Ologen™ is commercially available sterile material that surgeons can use to provide prompt surface reconstruction. These are the main strengths of this study. Ologen™ is currently used mainly in filtration surgery of glaucoma. There are a number of possible applications of OCM implants to ocular surface reconstruction such as scleral thinning, recurrent pterygium, and traumatic tissue defects. The patients’ perspective of OCM graft in this study was that both felt comfortable after reepithelialization, without ocular discomfort, ocular pain or severe foreign body sensation. Both were satisfied with their ocular surface cosmesis over the two years of observation.

In conclusion, our results suggest that the Ologen™ graft with CAU is potentially a new, safe, easy, and effective alternative treatment for ocular surface reconstruction, especially in scleromalacia or scleral necrosis after surgical excision of pterygium.

## Consent

Written informed consent was obtained from the patients for publication of this case report and any accompanying images. A copy of the written consent is available for review by the editor of this journal.

## Abbreviations

OCM: Ologen™ collagen matrix
CAU: Conjunctival autograft
MMC: Mitomycin C
